# Autologous Graft-versus-Tumor Effect: Reality or Fiction?

**DOI:** 10.1155/2016/5385972

**Published:** 2016-08-22

**Authors:** Luis F. Porrata

**Affiliations:** Division of Hematology, Department of Medicine, Mayo Clinic, 200 First Street SW, Rochester, MN 55905, USA

## Abstract

In contrast to allogeneic hematopoietic stem cell transplantation, the current dogma is not an evidence of graft-versus-tumor effect in autologous hematopoietic stem cell transplantation; thus, it is assumed that autologous hematopoietic stem cell transplantation only relies on the high-dose chemotherapy to improve clinical outcomes. However, recent studies argue in favor of the existence of an autologous graft-versus-tumor without the detrimental complications of graft-versus-host disease due to the nonspecific immune response from the infused donor alloreactive immune effector cells in allogeneic hematopoietic stem cell transplantation. Herein, this paper reviews the clinical evidence of an autologous graft-versus-tumor effect based on the autograft collected and infused host immune effector cells and host immunity recovery after autologous hematopoietic stem cell transplantation affecting clinical outcomes in cancer patients.

## 1. Introduction

The graft-versus-tumor effect [1] and donor lymphocyte infusion [2] have shifted our understanding from high-dose chemotherapy to the infused alloreactive donor immune effector cells as the underlying mechanism explaining the cancer curative potential of allogeneic hematopoietic stem cell transplantation. However, the immune response observed after allogeneic hematopoietic stem cell transplantation is not only tumor specific, as the donor alloreactive immune effector cells producing the graft-versus-tumor effect also result in graft-versus-host disease. On the other hand, the relapse rates reported after autologous hematopoietic stem cell transplantation are attributed to the assumption of the lack of graft-versus-tumor effect as seen in allogenic hematopoietic stem cell transplantation. In this paper, I will present clinical evidence that autologous graft-versus-tumor effect is a reality and no fiction.

## 2. Absolute Lymphocyte Count Recovery after Autologous Hematopoietic Stem Cell Transplantation

The simplest and less expensive immunological test is the absolute lymphocyte count obtained from the complete blood cell count. In allogeneic hematopoietic stem cell transplantation, early absolute lymphocyte count recovery, as a surrogate marker of immune reconstitution, is associated with prolonged survival [3, 4]. Our group analyzed the absolute lymphocyte count recovery at day 15 after autologous hematopoietic stem cell transplantation to assess if this biomarker has any prognostic ability to assess survival outcomes. We reported in patients treated with autologous hematopoietic stem cell transplantation for multiple myeloma and B-cell non-Hodgkin's lymphoma superior overall survival and progression-free survival if the absolute lymphocyte count at day 15 after autologous hematopoietic stem cell transplantation was equal to or greater than 500 cells/*µ*L compared with those whose absolute lymphocyte count at day 15 after autologous hematopoietic stem cell transplantation was less than 500 cells/*µ*L [5]. The absolute lymphocyte count at day 15 after autologous hematopoietic stem cell transplantation remained an independent prognostic factor for overall survival and progression-free survival by multivariate analysis for both conditions: B-cell non-Hodgkin's lymphoma and multiple myeloma.  Table 1 reports survival outcomes for multiple different cancers based on the absolute lymphocyte count recovery after autologous hematopoietic stem cell transplantation. To further validate the prognostic ability of the absolute lymphocyte count at day 15 after autologous hematopoietic stem cell transplantation to predict clinical outcomes, we conducted a prospective study. Our prospective study revealed a 5-year overall survival rates of 80% versus 37% and 5-year progression-free survival rates of 63% versus 13% for lymphoma patients with an absolute lymphocyte count equal to or greater than 500 cells/*µ*L compared with less than 500 cells/*µ*L at day 15 after autologous hematopoietic stem cell transplantation, respectively [6] (see Table 1). The European Group for Blood and Marrow Transplantation approved a multicenter prospective study entitled “Graft and Outcome in Autologous Stem Cell Transplantation: A Prospective GOA Study.” A recently published prospective study from the GOA confirmed in aggressive non-Hodgkin's lymphoma superior survival in patients with an absolute lymphocyte count equal to or greater than 500 cells/*µ*L at day 15 after autologous hematopoietic stem cell transplantation compared with those who did not [7] (see Table 1). The absolute lymphocyte count at day 15 after autologous hematopoietic stem cell transplantation as a surrogate marker of host immune recovery provides the first indirect clinical evidence of an autologous graft-versus-tumor effect.

## 3. Day 15 Immune Effector Cells after Autologous Hematopoietic Stem Cell Transplantation

A limitation of the absolute lymphocyte count at day 15 after autologous hematopoietic stem cell transplantation is that it does not specify what lymphocyte subset is conveying the survival benefit. To address this limitation we analyzed the lymphocyte subset recovery at day 15 after autologous hematopoietic stem cell transplantation. We identified natural killer cells as the key lymphocyte subset at day 15 after autologous hematopoietic stem cell transplantation affecting survival. Our study revealed 5-year overall survival rates of 76% versus 36% and 5-year progression-free survival rates of 57% versus 9% for non-Hodgkin's lymphoma patients with an absolute natural killer cell count equal to or greater than 80 cells/*µ*L compared with less than 80 cells/*µ*L at day 15 after autologous hematopoietic stem cell transplantation, respectively [6]. The significance of natural killer cell recovery after autologous hematopoietic stem cell transplantation was further confirmed in multiple myeloma patients, as better clinical outcomes were documented with higher natural killer cell recovery numbers [8]. Our group also reported that non-Hodgkin's lymphoma patients with higher expression of interleukin-15 at day 15 after autologous hematopoietic stem cell transplantation was associated with natural killer (NK) cell recovery and survival with a 5-year overall survival rates of 79% versus 47% and 5-year progression-free survival rates of 63% versus 31% for patients with an interleukin-15 equal to or greater than 76.5 pg/mL compared with less than 76.5 pg/mL at day 15 after autologous hematopoietic stem cell transplantation, respectively [9]. As maintenance therapy after autologous hematopoietic stem cell transplantation is currently undergoing clinical trials [10], our findings could provide a platform for the development of postautologous hematopoietic stem cell transplantation cytokine therapy to target specific host immunity effector cells to improve clinical outcomes in autologous hematopoietic stem cell transplantation.

Another immune effector cell factor that we identified as a negative prognostic factor for survival at day 15 after autologous hematopoietic stem cell transplantation is the absolute monocyte count. We identified that patients with an absolute monocyte count equal to or greater than 600 cells/*µ*L did worst compared with patients with an absolute monocyte count less than 600 cells/*µ*L: 5-year overall survival rates of 27% versus 74% and 5-year progression-free survival rates of 24% versus 68% for patients with an absolute monocyte count equal to or greater than 600 cells/*µ*L compared with less than 600 cells/*µ*L at day 15 after autologous hematopoietic stem cell transplantation, respectively [11]. There are currently no studies published analyzing the monocyte phenotype recovery after autologous hematopoietic stem cell transplantation. We are in the process of investigating if the monocytes recovery after autologous hematopoietic stem cell transplantation is monocytic myeloid-derived suppressor cells, as these types of immune effector cells have been implicated in the production of immunosuppressive cytokines, disruption of the class 1 major histocompatibility complex causing T-cells to become unresponsive to antigen-specific interaction, Fas-FasL interaction producing T-cell apoptosis, induction of regulatory T-cells, and inhibition of natural killer cells function and proliferation [12]. These immunosuppressive mechanisms of monocytic myeloid-derived suppressor cells could damper the benefits of an autologous graft-versus-tumor effect as they can directly affect natural killer cell function.

## 4. Autograft Lymphocytes as a Source of Absolute Lymphocyte Count Recovery in Autologous Hematopoietic Stem Cell Transplantation

The source of immune system recovery in recipients undergoing allogeneic hematopoietic stem cell transplantation can be directly traced and tested from the donor stem cells. In autologous hematopoietic stem cell transplantation, the sources of absolute lymphocyte count recovery can be divided into two groups: (1) the host and (2) the autograft [30]. From the host, sources of lymphocyte recovery include host stem cells and host lymphocytes surviving the high-dose chemotherapy. The host stem cells surviving high-dose chemotherapy most likely do not influence day 15 absolute lymphocyte count after autologous hematopoietic stem cell transplantation because without the infusion of stem cells prolonged myelosuppression is observed. To identify host lymphocytes surviving high-dose chemotherapy after autologous hematopoietic stem cell transplantation is difficult in comparison with allogeneic hematopoietic stem cell transplantation where the development of mixed chimerism in allogeneic hematopoietic stem cell transplantation allows discrimination of host-versus-donor lymphocytes. Such discrimination is not possible in autologous hematopoietic stem cell transplantation until the development of humans' marking studies of autograft lymphocytes.

The second source of absolute lymphocyte count recovery after autologous hematopoietic stem cell transplantation lies in the cells collected and infused from the autograft. The absolute lymphocyte count recovery after autologous hematopoietic stem cell transplantation could come from two autograft cell lines: (1) infused stem cells or (2) infused autograft lymphocytes. The autograft collection in our institution does not undergo any additional processing, such as T-cell depletion, beyond cryopreservation; thus, all cells collected in the autograft (stem cells and immune effector cells) are infused back to the patient. In order to understand the impact of the cells collected and infused from the autograft on postautologous hematopoietic stem cell transplantation on lymphocyte reconstitution (i.e., absolute lymphocyte count), we evaluated the impact of both autograft stem cells and autograft lymphocytes on the absolute lymphocyte count recovery after autologous hematopoietic stem cell transplantation. We identified no association between the amount of stem cells infused and day 15 absolute lymphocyte count after autologous hematopoietic stem cell transplantation. Nevertheless, a strong positive correlation was identified between the number of collected and infused autograft lymphocyte content and day 15 absolute lymphocyte count recovery after autologous hematopoietic stem cell transplantation. We named the autograft lymphocytes as autograft absolute lymphocyte count. Patients infused with higher numbers of autografts absolute lymphocyte count directly affected not only the day 15 absolute lymphocyte recovery but also clinical outcomes in patients with multiple myeloma and B-cell non-Hodgkin's lymphoma after autologous hematopoietic stem cell transplantation. Superior survival was observed in patients infused with an autograft absolute lymphocyte count ≥ 0.5 × 10^9^ lymphocytes/kg [31, 32]. This finding has been recently confirmed by other investigators in multiple myeloma patients treated with autologous hematopoietic stem cell transplantation [33]. To validate the significance of this discovery, we just recently reported a Phase III double blind clinical trial demonstrating that, in B-cell non-Hodgkin's lymphoma patients infused with an autograft absolute lymphocyte count ≥ 0.5 × 10^9^ lymphocytes/kg, experienced superior overall survival and progression-free survival compared with those who did not: 2-year overall survival rates of 95% versus 70%, *P* < 0.004 and 2-year progression-free survival of 79% versus 50%, *P* < 0.0025 [29]. These data support for the first time that the autograft absolute lymphocyte count is the biomarker producing an autograft graft-versus-tumor effect in patients undergoing autologous hematopoietic stem cell transplantation. Therefore, the autograft in autologous hematopoietic stem cell transplantation should be erroneously viewed not only for “bone marrow rescue” necessary for hematologic engraftment (i.e., recovery of white blood cells, red cells, and platelets) but also as an adoptive immunotherapeutic strategy whereas autograft immune effector cells directly influence cancer clinical outcomes. Our current clinical practice is changing to target not only enough stem cells for hematologic engraftment but also an autograft absolute lymphocyte count target of a value equal to or greater than 0.5 × 10^9^ lymphocytes/kg for immunologic engraftment and improve clinical outcomes after autologous hematopoietic stem cell transplantation.

## 5. Autograft Immune Effector Cells

The association between autograft absolute lymphocyte count and survival sets a platform to investigate the specific immune effector cells in the autograft for the development of immunotherapeutic strategies to improve clinical outcomes after autologous hematopoietic stem cell transplantation. Among the autograft immune effector cells showing an association with better clinical outcomes after autologous hematopoietic stem cell transplantation included T-cells (specifically CD4) [25], dendritic cells type 1 (cytotoxic) [24], and natural killer cells [29].  Table 2 depicts the survival outcomes based on the infused autograft immune effector cells. All these autograft immune effectors could be used as targets to enhance host immunity antitumor activity to improve survival [34].

In addition to the above immune effector cells, monocytes are also collected in the autograft and infused back to patients. As day 15 absolute monocyte count has been reported to be a negative prognostic factor for survival, similarly, autograft absolute monocyte count is a negative prognostic factor for clinical outcomes after autologous hematopoietic stem cell transplantation. We combine the autograft absolute lymphocyte count, as surrogate marker of host immunity, and the autograft absolute monocyte count, as a surrogate marker of tumor microenvironment, into an autograft lymphocyte to monocyte ratio [26–28]. Patient with a higher autograft lymphocyte to monocyte ratio experienced superior survival after autologous hematopoietic stem cell transplantation (see Table 2). This simple ratio can be used to manipulate the autograft collection to ensure a higher lymphocyte to monocyte ratio to improve survival after autologous hematopoietic stem cell transplantation.

## 6. Autologous Immunologic Graft Engineering

The relationship between autograft immune effector cells and autograft absolute lymphocyte count and clinical outcomes after autologous hematopoietic stem cell transplantation warrants the development of strategies to engineer an immunologic competent autograft by maximizing immune effector cells harvesting with direct impact on immunologic recovery and survival after autologous hematopoietic stem cell transplantation.

One strategy relies on the time interval from last chemotherapy to proceed with autologous stem cell transplantation. The collection of the autograft absolute lymphocyte count directly depends of the peripheral blood absolute lymphocyte count at the time of apheresis collection [31]. This implies that the more immunocompetent the patient is at the time of apheresis collection increases the probability of yielding higher autograft absolute lymphocyte count as the patient has enough time to recover from the immunosuppressive effect of the chemotherapy [35]. Our group reported that lymphoma patients with equal to or greater than 55-day interval from last chemotherapy experienced better overall and progression-free survival compared with lymphoma patient proceeding less than 55 days from last chemotherapy to autologous hematopoietic stem cell transplantation (median overall survival was not reached versus 21 months, *P* < 0.0008 and median progression-free survival of 76 months versus 9 months, *P* < 0.0025, resp.). The survival advantage between the groups of values equal to or greater than 55 days versus less the 55-day interval from last chemotherapy to autologous hematopoietic stem cell transplantation was attributed to the higher peripheral blood lymphocyte count at the time of apheresis collection leading to a higher collected and infused autograft absolute lymphocyte count translating into better clinical outcomes in the equal to or greater than 55 days versus less than 55 days groups [36]. Nevertheless, not all patients have the luxury to wait two months to allow for immune recovery from last chemotherapy to proceed with autologous stem cell transplantation. Therefore, other strategies are needed to maximize harvesting autologous immune effector cells.

A second strategy is to assess how the current stem cell mobilization regimens affect the collection of autologous immune effector cells (i.e., lymphocytes). Prior to the approval of Plerixafor for the mobilization of stem cells, the two stem cell mobilization modalities were the use of growth factor alone such as granulocyte-colony stimulation factor (G-CSF) and chemotherapy mobilization with the use of chemotherapy and G-CSF. We identified in multiple myeloma patients mobilized with G-CSF alone higher collection of autograft absolute lymphocyte count compared with multiple myeloma patients mobilized with G-SCF + Cytoxan chemotherapy (C + G-CSF: G-CSF = 0.764 × 10^9^ lymphocytes/kg (range: 0.146–1.803) versus C + G-CSF of 0.212 × 10^9^ lymphocytes/kg (range: 0.016–1.26), *P* < 0.0001. Due to the higher collection of autograft absolute lymphocyte count in the G-CSF groups, the G-SCF experienced better survival compared with the C + G-CSF [37]. This observation has been confirmed by Hiwase et al. [38], reporting also in multiple myeloma patients higher lymphocyte collection in the growth factor mobilized group versus growth factor and chemotherapy group. Superior survival was observed in the group that collected and infused higher number of autologous lymphocytes. Lymphoma patients were mobilized with chemoimmunotherapy (i.e., Rituximab); and growth factor versus chemotherapy and growth factors were associated with delayed lymphocyte recovery after autologous hematopoietic stem cell transplantation in the chemoimmunotherapy group [39]. These findings suggest that stem cell mobilization regimens using immunosuppressive agents such as Rituximab and chemotherapy are detrimental for the collection and infusion of autologous immune effector cells with direct impact on clinical outcomes after autologous hematopoietic stem cell transplantation.

G-CSF has been the backbone for stem cell mobilization. However, G-CSF has been associated with immunosuppressive effects such as the generation of myeloid-derived suppressor cells [40] and the generation of human T regulatory (Treg) cells [41]. To overcome the immunosuppressive side effects of G-CSF, another strategy is to combine G-CSF with agents to increase the proliferation and mobilization of autologous immune effector cells to collect and infuse besides stem cells to patients undergoing autologous hematopoietic stem cell transplantation. Plerixafor, a CXCR4 antagonist, has been approved to be used in combination with G-CSF for stem cell mobilization. In regard to immune effector cells, the CXCL12/CXCR4 has been identified as a chemokine signaling controlling T and NK cells trafficking, suggesting that Plerixafor could be used not only for stem cell mobilization but also as immune effector cells mobilization agent. Our group published that patients mobilized with Plerixafor achieved higher number of autograft absolute lymphocyte count, autograft CD3, CD4, CD8, and NK cells compared with control group that did not receive Plerixafor [42]. Recent studies have shown increased mobilization of T-cells (CD3, CD4, and CD8), NK cells, and dendritic cells in patients mobilized with Plerixafor [43, 44]. Thus, randomized studies comparing Plerixafor versus non-Plerixafor groups to assess autologous immune effector cells mobilization are warranted.

Since the infusion of autograft helper T-cells (CD4) and NK cells is associated with survival, cytokines specifically to target these immune effector cells are good candidates to test. In patients with breast cancer, Sosman et al. [45] demonstrated higher NK cell recovery at day 14 after autologous stem cell transplantation for patients mobilized with interleukin-2 and G-CSF compared with patients mobilized with G-CSF alone. Other cytokines candidates due to their NK cell proliferation effect to consider for specifically targeting autologous NK cells mobilization are interleukin-15 [46] and interleukin-21 [47]. Bruserud and Ulvestad [48] demonstrated in acute leukemia patients with treatment-induced cytopenias that interleukin-15 could also induce T-cell proliferation. This finding is significant because it makes interleukin-15 a more appealing cytokine to study for autologous immune effector cells mobilization as interleukin-15 targets the proliferation of both T-cells and NK cells. Interleukin-6 is another possible candidate as interleukin-6 increases CD4 survival and shifts CD4 differentiation to a helper T-cell (Th2) [49]. In addition, inteleukin-6 is a major inducer of interleukin-21 production in CD4. By upregulation of interleukin-21, interleukin-6 might enhance also NK cells proliferation [49]. Interleukin-7 has also been reported to increase T-cells proliferation (i.e., CD4 and CD8) from chemotherapy-induced leukopenia [50]. Furthermore, in patients with idiopathic CD4 lymphocytopenia, the administration of interleukin-7 resulted in the increase of circulating CD4 [51]. Interleukin-7 is important for thymopoiesis, T-cell homeostasis, and survival [52]. Thus, interleukin-7 could be considered for elderly patients undergoing autologous hematopoietic stem cell transplantation without a functional thymus as an individualized immune effector cell mobilization agent.

Another important factor in autograft immune engineering is cryopreservation to ensure that the infused autologous immune effector cells are viable cells. Our institution currently uses 10% dimethyl sulfoxide (DMSO) for cryopreservation. However, Akkök et al. [53] reported a comparison study of 5% versus 10% DMSO cryopreservation. The study identified no difference in regard to neutrophil and platelet engraftment. In addition, the day 15 absolute lymphocyte count recovery after autologous hematopoietic stem cell transplantation was the same between the 5% and 10% DMSO groups. An advantage of using 5% DMSO versus 10% DMSO is less graft volume, thus, allowing higher viable cell concentrations not only of stem cells but also of autologous immune effector cells.

## 7. Conclusion

The association between autograft absolute lymphocyte count, day 15 absolute lymphocyte count after autologous hematopoietic stem cell transplantation, and survival supports for the concept of an autologous graft-versus-tumor effect as the infusion of autograft lymphocytes has a direct impact not only on immune reconstitution but also on survival after autologous hematopoietic stem cell transplantation. In allogeneic hematopoietic stem cell transplantation, the strongest clinical evidence of a graft-versus-tumor effect comes from donor lymphocyte infusion. Similar argument can apply in the autologous hematopoietic stem cell transplantation as the autograft absolute lymphocyte count is another term for an autologous lymphocyte infusion with direct impact on clinical outcomes, thus, making an autologous graft-versus-tumor effect a reality and no fiction.

## Figures and Tables

**Table 1:** Survival outcomes based on absolute lymphocyte count recovery after autologous hematopoietic stem cell transplantation.

Study [reference]	Study design (disease)	Days after AHSCT ALC obtained	ALC cut-off value (*µ*/L)	OS			PFS		
				yrs	rates	*P*	yrs	rates	*P*
Porrata et al., 2001 [5]	Retrospective					<0.0001			<0.0001
	(non-Hodgkin's lymphoma)	15	≥500	5	85%		5	72%	
			<500	5	15%		5	0%	
	Retrospective					<0.0001			<0.0003
	(multiple myeloma)	15	≥500	5	30%		5	20%	
			<500	5	0%		5	0%	

Porrata et al., 2001 [13]	Retrospective					<0.0001			<0.0001
	(metastatic breast cancer)	15	≥500	3	55%		3	45%	
			<500	3	0%		3	0%	

Porrata et al., 2002 [14]	Retrospective					<0.0001			<0.002
	(Hodgkin's lymphoma)	15	≥500	5	80%		5	50%	
			<500	5	30%		5	20%	

Porrata et al., 2002 [15]	Retrospective					<0.0009			<0.0008
	(acute myelogenous leukemia)	15	≥500	5	68%		5	66%	
			<500	5	19%		5	5%	

Ferrandina et al., 2003 [16]	Retrospective					<0.0015			<0.0026
	(ovarian cancer)	365	≥850^*∗*^	3	93%		3	86%	
			<850	3	62%		3	23%	

Gordan et al., 2003 [17]	Retrospective					0.27			<0.02
	(Hodgkin's and non-Hodgkin's lymphoma)	15	≥667	5	70%		5	55%	
			<667	5	60%		5	25%	

Nieto et al., 2004 [18]	Retrospective					<0.04			<0.007
	(metastatic breast cancer)	15	≥500	5	42%		5	58%	
			<500	5	29%		5	18%	

Kim et al., 2004 [19]	Retrospective					<0.005			<0.011
	(T-cell non-Hodgkin's lymphoma)	25	≥1000	5	48%		5	80%	
			<1000	5	18%		5	30%	

Porrata et al., 2005 [20]	Retrospective					<0.0003			<0.0001
	(systemic amyloidosis)	15	≥500	5	95%		5	80%	
			<500	5	40%		5	30%	

Kim et al., 2006 [21]	Retrospective					<0.0156			<0.0243
	(multiple myeloma)	23	≥1000	5	50%		5	35%	
			<1000	5	27%		5	10%	

Boulassel et al., 2006 [22]	Retrospective					<0.001			<0.001
	(Lymphoproliferative disorders)	15	≥500	3	91%		5	70%	
			<500	3	68%		5	20%	

Joao et al., 2006 [23]	Retrospective					<0.01			<0.0006
	(mantle cell lymphoma)	15	≥500	5	70%		5	70%	
			<500	5	25%		5	5%	

Porrata et al., 2008 [6]	Prospective					<0.0001			<0.0001
	(non-Hodgkin's lymphoma)	15	≥500	5	80%		5	63%	
			<500	5	37%		5	13%	

Valtola et al., 2016 [7]	Prospective				<0.002				<0.015
	(non-Hodgkin's lymphoma)	15	≥500	3	88%		3	80%	
			<500	3	30%		3	38%	

^*∗*^This number is based on the CD3 count. ALC: absolute lymphocyte count; AHSCT: autologous hematopoietic stem cell transplantation; yrs: years.

**Table 2:** Autograft immune effector cells and survival after autologous hematopoietic stem cell transplantation.

Study [reference]	Disease (number of patients)	Autograft immune effector cells	2-year overall survival rates	2-year progression-free survival rates
Dean et al., 2005 [24]	Diffuse large B-cell lymphoma (53)	DC 1 ≥ 0.06 0.45 × 10^9^ cells/kg		55%
		DC 1 < 0.06 0.45 × 10^9^ cells/kg		35%
				(*P* < 0.04)

Schmidmaier et al., 2008 [25]	Multiple myeloma (41)	CD4 ≥ 0.45 × 10^9^ cells/kg		80%
		CD4 < 0.45 × 10^9^ cells/kg		40%
				(*P* < 0.003)

Porrata et al., 2014 [26]	Diffuse large B-cell lymphoma (379)	Lymphocyte to monocyte ratio ≥ 1.0	85%	75%
		Lymphocyte to monocyte ratio < 1.0	40%	30%
			(*P* < 0.0001)	(*P* < 0.0001)

Porrata et al., 2014 [27]	Hodgkin's lymphoma (183)	Lymphocyte to monocyte ratio ≥ 1.0	95%	85%
		Lymphocyte to monocyte ratio < 1.0	60%	30%
			(*P* < 0.0001)	(*P* < 0.0001)

Porrata et al., 2015 [28]	T-cell non-Hodgkin's lymphoma (109)	Lymphocyte to monocyte ratio ≥ 1.0	90%	80%
		Lymphocyte to monocyte ratio < 1.0	45%	30%
			(*P* < 0.0001)	(*P* < 0.0001)

Porrata et al., 2016 [29]	Non-Hodgkin's lymphoma (122)	NK ≥ 0.06 0.45 × 10^9^ cells/kg	85%	79%
		NK < 0.06 0.45 × 10^9^ cells/kg	70%	45%
			(*P* = 0.06)	(*P* < 0.02)

DC: dendritic cells; NK: natural killer cells.

## References

[1] Gill S., Porter D. L. (2013). Reduced-intensity hematopoietic stem cell transplants for malignancies: harnessing the graft-versus-tumor effect. *Annual Review of Medicine*.

[2] Chang Y.-J., Huang X.-J. (2013). Donor lymphocyte infusions for relapse after allogeneic transplantation. When, if and for whom?. *Blood Reviews*.

[3] Powles R., Singhal S., Treleaven J., Kulkarni S., Horton C., Mehta J. (1998). Identification of patients who may benefit from prophylactic immunotherapy after bone marrow transplantation for acute myeloid leukemia on the basis of lymphocyte recovery early after transplantation. *Blood*.

[4] Pavletic Z. S., Joshi S. S., Pirruccello S. J. (1998). Lymphocyte reconstitution after allogeneic blood stem cell transplantation for hematologic malignancies. *Bone Marrow Transplantation*.

[5] Porrata L. F., Gertz M. A., Inwards D. J. (2001). Early lymphocyte recovery predicts superior survival after autologous hematopoietic stem cell transplantation in multiple myeloma or non-Hodgkin lymphoma. *Blood*.

[6] Porrata L. F., Inwards D. J., Ansell S. M. (2008). Early lymphocyte recovery predicts superior survival after autologous stem cell transplantation in non-hodgkin lymphoma: a prospective study. *Biology of Blood and Marrow Transplantation*.

[7] Valtola J., Varmavuo V., Ropponen A. (2016). Early immune recovery after autologous transplantation in non-Hodgkin lymphoma patients: predictive factors and clinical significance. *Leukemia and Lymphoma*.

[8] Rueff J., Medinger M., Heim D., Passweg J., Stern M. (2014). Lymphocyte subset recovery and outcome after autologous hematopoietic stem cell transplantation for plasma cell myeloma. *Biology of Blood and Marrow Transplantation*.

[9] Porrata L. F., Inwards D. J., Micallef I. N. (2010). Interleukin-15 affects patient survival through natural killer cell recovery after autologous hematopoietic stem cell transplantation for non-Hodgkin lymphomas. *Clinical and Developmental Immunology*.

[10] Taverna J. A., Yun S. G., Jonnadula J. (2016). Role of maintenance therapy after high-dose chemotherapy and autologous hematopoietic cell transplantation in aggressive lymphomas: a systemic review. *Biology of Blood and Marrow Transplantation*.

[11] Porrata L. F., Inwards D. J., Ansell S. M., Micallef I. N. (2011). Day 15 peripheral blood lymphocyte/monocyte ratio post-autologous peripheral hematopoietic stem cell transplantation and survival in diffuse large B-cell lymphoma. *Journal of Stem Cell Research & Therapy*.

[12] Ansell K., Porrata L. F. (2013). Autograft monocytes: the bad humors of autologous peripheral blood hematopoietic stem cell transplantation. *Journal of Stem Cell Research & Therapy*.

[13] Porrata L. F., Ingle J. N., Litzow M. R., Geyer S., Markovic S. N. (2001). Prolonged survival associated with early lymphocyte recovery after autologous hematopoietic stem cell transplantation for patients with metastatic breast cancer. *Bone Marrow Transplantation*.

[14] Porrata L. F., Inwards D. J., Micallef I. N., Ansell S. M., Geyer S. M., Markovic S. N. (2002). Early lymphocyte recovery post-autologous haematopoietic stem cell transplantation is associated with better survival in Hodgkin's disease. *British Journal of Haematology*.

[15] Porrata L. F., Litzow M. R., Tefferi A. (2002). Early lymphocyte recovery is a predictive factor for prolonged survival after autologous hematopoietic stem cell transplantation for acute myelogenous leukemia. *Leukemia*.

[16] Ferrandina G., Pierelli L., Perillo A. (2003). Lymphocyte recovery in advanced ovarian cancer patients after high-dose chemotherapy and peripheral blood stem cell plus growth factor support: clinical implications. *Clinical Cancer Research*.

[17] Gordan L. N., Sugrue M. W., Lynch J. W., Williams K. D., Khan S. A., Moreb J. S. (2003). Correlation of early lymphocyte recovery and progression-free survival after autologous stem-cell transplant in patients with Hodgkin's and non-Hodgkin's lymphoma. *Bone Marrow Transplantation*.

[18] Nieto Y., Shpall E. J., McNiece I. K. (2004). Prognostic analysis of early lymphocyte recovery in patients with advanced breast cancer receiving high-dose chemotherapy with an autologous hematopoietic progenitor cell transplant. *Clinical Cancer Research*.

[19] Kim H., Sohn H.-J., Kim S.-E. (2004). Lymphocyte recovery as a positive predictor of prolonged survival after autologous peripheral blood stem cell transplantation in T-cell non-Hodgkin's lymphoma. *Bone Marrow Transplantation*.

[20] Porrata L. F., Gertz M. A., Litzow M. R. (2005). Early lymphocyte recovery predicts superior survival after autologous hematopoietic stem cell transplantation for patients with primary systemic amyloidosis. *Clinical Cancer Research*.

[21] Kim H., Sohn H.-J., Kim S., Lee J.-S., Kim W.-K., Suh C. (2006). Early lymphocyte recovery predicts longer survival after autologous peripheral blood stem cell transplantation in multiple myeloma. *Bone Marrow Transplantation*.

[22] Boulassel M. R., Herr A. L., deB Edwards M. D. (2006). Early lymphocyte recovery following autologous peripheral stem cell transplantation is associated with better survival in younger patients with lymphoproliferative disorders. *Hematology*.

[23] Joao C., Porrata L. F., Inwards D. J. (2006). Early lymphocyte recovery after autologous stem cell transplantation predicts superior survival in mantle-cell lymphoma. *Bone Marrow Transplantation*.

[24] Dean R., Masci P., Pohlman B. (2005). Dendritic cells in autologous hematopoietic stem cell transplantation for diffuse large B-cell lymphoma: graft content and post transplant recovery predict survival. *Bone Marrow Transplantation*.

[25] Schmidmair R., Oversohl N., Schnabel B. (2008). Helper T cells (CD3+/CD4+) within the autologous peripheral blood stem cell graft positively correlate with event free survival of multiple myeloma patients. *Experimental Oncology*.

[26] Porrata L. F., Inwards D. J., Ansell S. M. (2014). Infused autograft lymphocyte to monocyte ratio and survival in diffuse large B cell lymphoma. *Biology of Blood and Marrow Transplantation*.

[27] Porrata L. F., Inwards D. J., Ansell S. M. (2015). Infused autograft lymphocyte to monocyte ratio predicts survival in classical Hodgkin lymphoma. *Journal of Blood Medicine*.

[28] Porrata L. F., Inwards D. J., Ansell S. M. (2015). Infused autograft lymphocyte-to-monocyte ratio and survival in T-cell lymphoma post-autologous peripheral blood hematopoietic stem cell transplantation. *Journal of Hematology & Oncology*.

[29] Porrata L. F., Burgstaler E. A., Winters J. L. (2016). Immunologic autograft engineering and survival in non-Hodgkin lymphoma. *Biology of Blood and Marrow Transplantation*.

[30] Porrata L. F., Markovic S. N. (2004). Timely reconstitution of immune competence affects clinical outcome following autologous stem cell transplantation. *Clinical and Experimental Medicine*.

[31] Porrata L. F., Lotzow M. R., Inwards D. J. (2004). Infused peripheral blood autograft absolute lymphocyte count correlates with day 15 absolute lymphocyte count and clinical outcome after autologous peripheral hematopoietic stem cell transplantation in non-Hodgkin's lymphoma. *Bone Marrow Transplantation*.

[32] Porrata L. F., Gertz M. A., Geyer S. M. (2004). The dose of infused lymphocytes in the autograft directly correlates with clinical outcome after autologous peripheral blood hematopoietic stem cell transplantation in multiple myeloma. *Leukemia*.

[33] Hiwase D. K., Hiwase S., Bailey M., Bollard G., Schwarer A. P. (2008). Higher infused lymphocyte dose predicts higher lymphocyte recovery, which in turn, predicts superior overall survival following autologous hematopoietic stem cell transplantation for multiple myeloma. *Biology of Blood and Marrow Transplantation*.

[34] Porrata L. F., Markovic S. N. (2010). Autograft mediated adoptive immunotherapy of cancer in the context of autologous stem cell transplantation. *World Journal of Clinical Oncology*.

[35] Mackall C. L., Fleisher T. A., Brown M. R. (1995). Age, thymopoiesis, and CD4+ T-lymphocyte regeneration after intensive chemotherapy. *The New England Journal of Medicine*.

[36] Holtan S. G., Porrata L. F., Inwards D. J. (2006). Timing of autologous stem cell transplantation from last chemotherapy affects lymphocyte collection and survival in non-Hodgkin lymphoma. *British Journal of Haematology*.

[37] Porrata L. F., Hayman S. R., Gertz M. A. (2005). The role of stem cell mobilization regimen on lymphocyte collection yield and survival after autologous hematopoietic stem cell transplantation in multiple myeloma. *Blood*.

[38] Hiwase D. K., Hiwase S., Bailey M., Bollard G., Schwarer A. P. (2008). The role of stem cell mobilization regimen on lymphocyte collection yield in patients with multiple myeloma. *Cytotherapy*.

[39] Kim M. K., Kim S., Lee S. S. (2007). Rituximab-ESHAP as a mobilization regimen for relapsed or refractory B-cell lymphomas: a comparison with ESHAP. *Transfusion*.

[40] Luyckx A., Schouppe E., Rutgeerts O. (2012). G-CSF stem cell mobilization in human donors induces polymorphonuclear and mononuclear myeloid-derived suppressor cells. *Clinical Immunology*.

[41] Rutella S., Bonanno G., Pierelli L. (2004). Granulocyte colony-stimulating factor promotes the generation of regulatory DC through induction of IL-10 and IFN-*α*. *European Journal of Immunology*.

[42] Holtan S. G., Porrata L. F., Micallef I. N. M. (2007). AMD3100 affects autograft lymphocyte collection and progression-free survival after autologous stem cell transplantion in non-Hodgkin lymphoma. *Clinical Lymphoma & Myeloma*.

[43] Gazitt Y., Freytes C. O., Akay C., Badel K., Calandra G. (2007). Improved mobilization of peripheral blood CD34^+^ cells and dendritic cells by AMD3100 plus granulocyte-colony-stimulating factor in non-Hodgkin's lymphoma patients. *Stem Cells and Development*.

[44] Gaugler B., Arbez J., Legouill S. (2013). Characterization of peripheral blood stem cell grafts mobilized by granulocyte colony-stimulating factor and plerixafor compared with granulocyte colony-stimulating factor alone. *Cytotherapy*.

[45] Sosman J. A., Stiff P., Moss S. M. (2001). Pilot trial of interleukin-2 with granulocyte colony stimulating factor for the mobilization of progenitor cells in advanced breast cancer patients undergoing high-dose chemotherapy: expansion of immune effectors within the stem-cell graft and post-stem-cell infusion. *Journal of Clinical Oncology*.

[46] Fehniger T. A., Caligiuri M. A. (2001). Interleukin 15: biology and relevance to human disease. *Blood*.

[47] Parrish-Novak J., Dillon S. R., Nelson A. (2000). Interleukin 21 and its receptor are involved in NK cell expansion and regulation of lymphocyte function. *Nature*.

[48] Bruserud Ø., Ulvestad E. (2000). Cytokine responsiveness of mitogen-activated T cells derived from acute leukemia patients with chemotherapy-induced leukopenia. *Journal of Interferon & Cytokine Research*.

[49] Dienz O., Rincon M. (2009). The effects of IL-6 on CD4 T cell responses. *Clinical Immunology*.

[50] Wendelbo Ø., Glenjen N., Bruserud Ø. (2002). Interleukin-7 (IL-7) in patients receiving intensive chemotherapy for acute myelogenous leukemia: studies of systemic IL-7 levels and IL-7 responsiveness of circulating T lymphocytes. *Journal of Interferon & Cytokine Research*.

[51] Sheikh V., Porter B. O., DerSimonian R. (2016). Administration of interleukin-7 increases CD4 T cells in idiopathic CD4 lymphocytopenia. *Blood*.

[52] Fry T. J., Mackall C. L. (2002). Interleukin-7: from bench to clinic. *Blood*.

[53] Akkök Ç. A., Liseth K., Nesthus I. (2008). Autologous peripheral blood progenitor cells cryopreserved with 5 and 10 percent dimethyl sulfoxide alone give comparable hematopoietic reconstitution after transplantation. *Transfusion*.

